# Draft Genome Sequences of 18 Psychrotolerant and 2 Thermotolerant Strains Representative of Particular Ecotypes in the *Bacillus cereus* Group

**DOI:** 10.1128/genomeA.01568-16

**Published:** 2017-02-02

**Authors:** Marie-Hélène Guinebretière, Valentin Loux, Véronique Martin, Pierre Nicolas, Vincent Sanchis, Véronique Broussolle

**Affiliations:** a UMR408 Sécurité et Qualité des Produits d’Origine Végétale, INRA, Université d’Avignon, Avignon, France; b MaIAGE, Mathématiques et Informatique Appliquées du Génome à l’Environnement, INRA, Université Paris-Sarclay, Jouy-en-Josas, France; c MICALIS Institute, INRA, AgroParisTech, Université Paris-Sarclay, Jouy-en-Josas, France

## Abstract

Bacteria from the* Bacillus cereus* group exhibit genetic and physiological diversity through different ecotypes. Here, we present the draft genome sequences of 20 bacterial strains belonging to the contrasted psychrotolerant and thermotolerant ecotypes.

## GENOME ANNOUNCEMENT

Over the past decade, advances in the understanding of phylogeny of the *Bacillus cereus* group (*B. cereus sensu lato*) showed a species complex including the food-poisoning *B. cereus sensu stricto* and *B. cytotoxicus*; *B. thuringiensis*, known as an insect pathogen; the “category A” pathogen *B. anthracis*; and the remaining *B. weihenstephanensis*, *B. mycoides*, and *B. pseudomycoides* species (1). More recently, a few new singletons have also been described (2).

In 2008, a complete ecotype structure was described and directly linked to the phylogenetic structure of the *B. cereus* group (3), indicating a probable weighty role of temperature and thermal niches on the evolutionary history of the whole *B. cereus* group. This phylogenetic and ecotypic structure, distributed into seven major phylogenetic subdivisions (designated by Roman numerals I to VII), was later shown to be coherent with multilocus sequence type clades characterized in the group (1) and was recently supported by phylogenomics (4). In this structure, two phylogenetic groups were identified as psychrotolerant thermotypes (groups II and VI), and one rare and distant group was identified as moderately thermotolerant (group VII, *B. cytotoxicus*). To enhance our knowledge of adaptation mechanisms toward psychrotolerance and thermotolerance in the *B. cereus* group, the genome of 18 psychrotolerant and two thermotolerant strains were sequenced. Numerous related genomes were already deposited in databanks over the past 10 years, but most of them were from mesophilic strains and very few from psychrotolerant and thermotolerant strains. Psychrotolerance determines the capacity of bacteria to potentially lead to food poisoning after ingestion of contaminated cold-stored products.

High-quality genomic DNA was obtained from purified isolates of each strain and used to generate Illumina libraries with a 350-bp insert gel size selection, according to an improved Fasteris *de novo* protocol (Fasteris SA, Plan-les-Ouates, Switzerland). An additional library was obtained for four strains using the Illumina Nextera Mate-Pair (3-kb insert gel size selection). Libraries were sequenced using HiSeq high-output DNA sequencing with paired-end reads of 2 × 100 bp and a final average sequence coverage of 130×. A *de novo* assembly was performed using Velvet version 1/2/08 (5). Contigs and scaffolds were organized and arranged using Mauve (6) and several complete genomes as references (E33L, ATCC 14579, and KBAB4). The 20 assemblies yielded 50 contigs/scaffolds on average, consisting of 4.1/4.2 Mb for the thermotolerant strains to 5.0/6.0 Mb for the psychrotolerant strains, with a mean *N*_50_ of 566,326 bp. Compared to the Fasteris *de novo* protocol alone, the Nextera mate-pair protocol did not significantly improve quality and coverage when supplemented.

Automatic annotation for each genome utilized the pipeline AGMIAL (7). Each genome is available in NCBI, and raw data can be provided upon request. As pXO1-like and pXO2-like plasmids are widely distributed in environmental isolates of *B. cereus sensu lato* and seem to play a role in ecotypic and pathotypic differentiation, their presence was predicted (2), as well as the *cry* insecticidal genes usually found in *B. thuringiensis* (8). Strain characteristics are described in Table 1. In-depth comparative analyses of these and other genomes are underway.

**TABLE 1:** Strain characteristics^a^

*B. cereus sensu lato* species, strain name	Phylogenetic group (ecotype)	Range of growth temperature (°C)	Sample source	Provided by	Assembly size (Mb)	No. of contigs	Sequencing depth (×)	pXO1-like or pXO2-like^b^	No. of predicted *cry* genes^c^	DDBJ/ENA/GenBank accession no.
*B. thuringiensis*, IEBC_T61001	II	7–40	Plant, Scotch pine	INRA, France	5.9	73	43	—	3	FMBI00000000
*B. cereus*, INRA Bc05-F1	II	7–40	Soil, France	INRA, France	5.7	20	158	—	1	FMBE00000000
*B. cereus*, INRA_SA'	II	8–40	Soil, France	INRA, France	5.6	74	76	—	—	FMJI00000000
*B. cereus*, INRA_ZB5-J	II	8–40	Courgette, France	INRA, France	6.1	89	120	pXO1-like	1	FMBH00000000
*B. cereus*, NVH 0674-98	II	8–40	Mashed swedes, Norway^d^	NVH, Norway	5.2	40	92	—	—	FMJM00000000
*B. cereus*, NVH 0861-00	II	8–40	Ice cream, Norway^d^	NVH, Norway	5.7	34	536	—	2	FMBJ00000000
*B. cereus*, RIVM_BC485	II	8–40	Chicken ragout, The Netherlands^d^	RIVM, The Netherlands	6.0	45	87	—	—	FMSK00000000
*B. cereus*, RIVM_BC120	II	7–40	Human feces^d^	RIVM, The Netherlands	5.7	39	110	—	—	FMIJ00000000
*B. cereus*, RIVM_BC126	II	7–40	Human feces^d^	RIVM, The Netherlands	5.4	39	154	—	—	FMJJ00000000
*B. cereus*, RIVM_BC938	II	7–40	Lamb’s lettuce/corn salad, The Netherlands^d^	RIVM, The Netherlands	5.9	117	75	pXO1-like	—	FMJO00000000
*B. cereus*, WSBC_10311	II	7–40	Soil, 1997	TUM, Germany	5.8	33	102	—	—	FMBG00000000
*B. cereus*, F2404B-79	II	9–40	Diarrheal food poisoning outbreak^d^	PHLS, England	5.4	59	120	—	—	FMJG00000000
*B. cereus*, NVH_141/1-01_V_C53	V	10–40	Vegetarian pasta, Norway^d^	NVH, Norway	5.6	83	49	pXO2-like	—	FMJK00000000
*B. weihenstephanensis*, INRA 5	VI	5–37	Vegetable purée, France	INRA, France	5.7	36	188	—	1	FLZU00000000
*B. cereus*, ADRIA_I21	VI	7–37	Raw carrots, France	ADRIA, France	5.6	60	139	—	—	FMJF00000000
*B. weihenstephanensis*, INRA_SL'	VI	7–37	Soil, France	INRA, France	4.9	33	92	—	—	FMJH00000000
*B. weihenstephanensis*, SDA_GO95	VI	7–37	Raw milk, Sweden	SDA, Sweden	5.4	92	129	pXO2-like	—	FMAK00000000
*B. weihenstephanensis*, SDA_NFFE664	VI	5–37	Dairy environment, Sweden	SDA, Sweden	5.2	56	192	pXO2-like	—	FMBF00000000
*B. cytotoxicus*, AFSSA_08CEB44bac	VII	20–50	Cooked semolina, France^d^	ANSES, France	4.1	80	133	—	1	FMIK00000000
*B. cytotoxicus*, NVH_883-00	VII	20–50	Spices, Norway	NVH, Norway	4.2	59	116	—	—	FMJN00000000

^a^ ADRIA, Association pour le Développement et la Recherche des Industries Alimentaires, Villers Bocage, France; AFSSA, ANSES, Agence Nationale de Sécurité Sanitaire de l’Alimentation, de l’Environnement et du Travail, Maison-Alfort, France; IEBC, Unité des Bactéries Entomopathogènes, Institut Pasteur, Paris, France; INRA, Institut National de Recherche Agronomique, Avignon or Jouy-en-Josas, France; NVH, the Norwegian School of Veterinary Science, Oslo, Norway; PHLS, Public Health Laboratory Service, London, United Kingdom; RIVM, Rijksinstituut voor Volksgezondheid en Milieu, Bilthoven, The Netherlands; SDA, Swedish Dairy Association, Lund, Sweden; TUM, Technische Universität München, Munich, Germany; WSBC, Weihenstephan *Bacillus cereus* Collection, Weihenstephan, Germany.

^b^ pXO1-like or pXO2-like plasmid prediction based on the presence of the *repX* or *repS* genes, respectively, and absence of anthrax virulence genes (2). —, not detected.

^c^ Predicted *cry* genes indicating the putative production of insecticidal toxins (8). —, not detected.

^d^ Diarrheal food poisoning strain.

### Accession number(s).

Genome accession numbers to public databases are listed in Table 1.
